# Overexpression of miR-146a-5p and miR-221-3p in Human Synovial MSC-like Cells Favoured the Expression of Pro-Inflammatory Mediators in an In Vitro Model of Rheumatoid Arthritis

**DOI:** 10.3390/cells15080691

**Published:** 2026-04-14

**Authors:** Melissa Payet, Matthieu Daniel, Brice Nativel, Franck Ah-Pine, Philippe Gasque, Xavier Guillot

**Affiliations:** 1 Research Unit ‘Etudes Pharmaco-Immunologique’ UR EPI, Université de la Réunion, 97400 Saint-Denis, France; 2 Service d’Anatomie et Cytologie Pathologiques, CHU de La Réunion Site SUD, 97400 Saint Pierre, France; 3 Immunology Laboratory (LICE-OI), CHU de La Réunion, 97400 Saint-Denis, France; 4 Rheumatology Department, CHU de La Réunion Site Félix Guyon, Allée des Topazes, CS 11021, 97400 Saint-Denis, France

**Keywords:** micro-RNAs, rheumatoid arthritis, inflammation, synovial tissue-derived mesenchymal stem cells

## Abstract

**Highlights:**

**What are the main findings?**

- MiR-146a-5p and miR-221-3p were upregulated in the plasma of patients with rheumatoid arthritis.
- MiR-146a-5p and miR-221-3p were also upregulated in synovial mesenchymal stem cells in response to pro-inflammatory mediators and may contribute to promoting inflammation.

**What are the implications of the main findings?**

- MiR-146a-5p and miR-221-3p could serve as potential biomarkers for inflammation in rheumatoid arthritis.
- Targeting miR-146a-5p and miR-221-3p may represent a novel therapeutic strategy to modulate inflammatory processes in rheumatoid arthritis.

**Abstract:**

In rheumatoid arthritis (RA), activated synovial tissue-derived mesenchymal stem cells (MSC) acquire a pathogenic phenotype and produce pro-inflammatory cytokines, chemokines, metalloproteinases, pro-osteoclastic and pro-angiogenic factors. The acquisition of this aggressive phenotype might be due to modified expression of micro-RNAs. We aimed to clarify the role of specific micro-RNAs (miR-146a-5p, miR-221-3p, miR-34a-3p, miR-150, miR-203a-3p and miR-155-3p) in an in vitro model of RA. Methods: Micro-RNA expression was determined in RA patient plasma and in commercial human synovial tissue-derived MSC-like cells stimulated with a panel of pro-inflammatory mediators (poly I:C, TNF-α, IL-1β, IFN-γ) to mimic the rheumatoid arthritis pathogenic setting. Next, unstimulated cells or TNF-α stimulated cells were transfected with miR-146a-5p mimic or miR-221-3p mimic. Protein and/or mRNA expressions of chemokines, cytokines, VEGF, MMPs and RANKL were determined by ELISA or qRT-PCR. MiR-34a-3p, miR-146a-5p, miR-150, miR-221-3p and miR-203a-5p were upregulated in RA patient plasma versus healthy controls. Moreover, synovial tissue-derived MSC-like cells expressed miR-146a-5p and miR-221-3p in response to pro-inflammatory mediators. Overexpression of miR-146a-5p increased CCL2 and CXCL8 expression and miR-221-3p increased IL-1β and IL-6 expression in synovial tissue-derived MSC-like cells stimulated with TNF-α. Conclusion: Overexpression of miR-146a-5p and miR-221-3p might favour inflammation and participate in rheumatoid arthritis pathogenesis.

## 1. Introduction

Rheumatoid arthritis (RA) is characterized, on a physiopathological level, by chronic inflammation, cartilage degradation and bone erosion [1]. Synovial tissue-derived mesenchymal stem cells (MSC) participate to RA pathogenesis [2,3]. These cells can be activated by DAMPs (Danger-Associated Molecular Patterns) or PAMPs (Pathogen-Associated Molecular Patterns) and acquire an aggressive and pathogenic phenotype leading to the production of pro-inflammatory mediators (e.g., IL-1β, TNF-α), specific chemokines (e.g., CCL2 (Chemokine ligand 2), CXCL8 (CXC motif chemokine ligand 8)) [4,5], metalloproteinases (MMPs) (e.g., MMP-1, MMP-3) [4,6] and pro-osteoclastic factors such as RANKL (Receptor Activator of NF-κB Ligand) [7,8]. Activated synovial tissue-derived MSC-like cells also produce VEGF (Vascular Endothelial Growth Factor) involved in angiogenesis [9,10,11].

In this context, synovial fibroblasts were considered in our study as synovial tissue-derived MSC-like cells. Several studies have supported the concept that synovial fibroblasts can be considered as synovium-derived MSCs, as they share phenotypic and functional characteristics with classical MSC populations [12,13]. Moreover, synovial fibroblasts have been described as immune-regulatory cells, similarly to MSCs [14]. We previously demonstrated that these cells express indoleamine 2,3-dioxygenase (*IDO*) under pro-inflammatory conditions, a functional property commonly described in MSC-like cells’ biology [15]. Taken together, these data support the use of synovial fibroblast populations as synovial tissue-derived MSC-like cells in our experimental model.

This phenotypic reprogramming of synovial tissue-derived MSC-like cells under the effect of molecular danger signal might be associated with an epigenetic reprogramming, including modified expression of a specific panel of miRs. Our team has also demonstrated the importance of epigenetic mechanisms, including miRs, DNA methylation, and histone modifications, in the regulation and pathophysiology of RA [16].

Micro-RNAs (miRs) are small non-coding RNAs which negatively regulate gene expression [17]. MiRs could represent potential therapeutic targets in chronic arthritis. Indeed, miRs can exert pro- or anti-inflammatory effects by inhibiting or favouring gene expression of pro- or anti-inflammatory mediators [18].

For instance, the expression of miR-146a can be induced by pro-inflammatory stimuli such as IL-1β and TNF-α [19]. In addition, the expression of miR-221-3p is upregulated in response to TNF-α. Within a pro-inflammatory microenvironment, activation of *NF-κB* signalling by IL-6 or TNF-α results in the upregulation of miR-34a expression [20].

Moreover, administration of a miR-34a inhibitor in a murine model reduces the expression levels of pro-inflammatory cytokines, including *TNF-α*, *IL-1β*, *IL-6*, and *IFN-γ*, in both synovial tissue and serum [21].

Moreover, altered expression of miRs might be responsible for the aggressive phenotype of synovial tissue-derived MSC-like cells in RA. Indeed, differential expression of miRs affected several intracellular pathways of synovial tissue-derived MSC-like cells involved in inflammation, bone erosion and cartilage degradation [22].

For example, overexpression of miR-146a has been shown to reduce the expression of pro-inflammatory cytokines through modulation of the *TLR4/NF-κB* signalling pathway [23]. Similarly, inhibition of miR-221 results in decreased expression of pro-inflammatory mediators, including *IL-1β* and *TNF-α* [24]. In synovial cells, overexpression of miR-155 has been reported to decrease the expression of pro-inflammatory cytokines, such as *IL-1β* and *TNF-α*, via downregulation of *FOXO3a* [25].

Overexpression of miR-203 has been reported to have increased expression of MMP-1 and IL-6 possibly through *NF-κB* signalling pathway in synovial cells [26]. The use of exosomes containing miR-150-5p on synovial cells resulted in the downregulation of *VEGF* and *MMP14* expression, as well as in the inhibition of synovial MSC-like cell proliferation and invasion [27].

Herein, we aimed to assess the expression of specific miRs in synovial tissue-derived MSC-like cells and RA patient plasma compared to patients free of inflammatory disease. We then sought to clarify the role of these miRs in the pathogenesis of RA by investigating the association between the expression of these miRs and the activation of cellular and molecular pathways known to represent biological signatures of RA.

## 2. Materials and Methods

### 2.1. Patients and Control Samples

All RA patients fulfilled the American College of Rheumatology criteria for the classification of RA. RA patients were recruited from CHU de La Reunion rheumatology department. Control samples of HC were collected from a group of patients admitted to the emergency ward between 24 December 2022 and 24 April 2023 for minor traumatic pathologies and not presenting systemic inflammatory response syndrome (non-severe peripheral traumatisms were confirmed by the absence of bone fracture on radiological examinations and an absence of inflammatory syndrome on biological parameters). This control group was investigated for comparative purposes. Samples of RA patients were collected between 27 October 2020 and 14 February 2023. Peripheral blood samples were collected from RA patients (*n* = 9) and healthy donors (*n* = 10). The demographic and clinical characteristics of RA patients and HC pare summarized in Table 1.

Peripheral blood (3 mL) of RA patients and HC was collected in EDTA tubes. Plasma was isolated on the day of blood collection by centrifugation at room temperature for 5 min at 2000 g. The plasma was carefully transferred to avoid disturbing the cellular layer and aliquoted into 350 µL fractions. Aliquots were stored at −90 °C until further analysis. Each aliquot was thawed only once immediately before miR extraction to avoid repeated freeze–thaw cycles. miR extraction was performed in March 2023.

This study was conducted in accordance with the French MR-004 methodology (research not involving human subjects but using human samples and associated data) and the Declaration of Helsinki. Biological samples and associated data were stored and used in accordance with the French regulations and the ethical charter of tumour banks of the French National Cancer Institute (INCa). Samples were obtained from the Biological Resource Center (CRB) of CHU de La Réunion (French Ministry of Research authorization number: AC-2022-4899), which ensures regulatory compliance of sample collection, storage and use. Informed consent was obtained from all participants.

### 2.2. Cell Culture Reagents

Primary culture of human synovial fibroblasts was purchased from Sciencell Research Laboratories (ScienceCell, 4700 Clinisciences, Carlsbad, CA, USA). Cells were cultured in Modified Eagle’s Medium as previously described [28]. Cells were maintained in a humid atmosphere with 5% of CO_2_.

### 2.3. Cell Treatments 

Human synovial tissue-derived MSC-like cells were placed in 6-well plate and maintained at 37 °C in a humid atmosphere with 5% of CO_2_. The medium was replaced twice a week. Cells were stimulated with Poly I:C (10 µg/mL), IL-1β, TNF-α, IFN-γ, TGF-β1 and PDGF-BB at 20 ng/mL during 6 h or 24 h.

### 2.4. Cell Transfection and Stimulation with TNF-α

MiR-146a-5p mimic (sense 5′-UGAGAACUGAAUUCCAUGGGUU-3′) and miR-221-3p mimic (sense 5′-AGCUACAUUGUCUGCUGGGUUUC-3′) were synthesized by GeneCust, Boynes, France. Synovial tissue-derived MSC-like cells were cultured in 12-well plate and at 80% confluency were transfected by miR-146a-5p and miR-221-3p (100 nM) using lipofectamine 3000 reagent (Invitrogen, L3000-008, Thermo Fisher Scientific, Waltham, MA, USA). Cells were incubated during 48 h in a humid atmosphere with 5% of CO_2_. After 6 h, the medium was replaced. Then, after 48 h of incubation, transfected cells were stimulated with TNF-α (20 ng/mL) during 6 h.

### 2.5. RNA Extraction and qRT-PCR

Total RNA was directly extracted from cultured cells using the Quick-RNA™ Viral Kit (Zymo, Ozyme, Irvine, CA, USA, cat. No. R1035) according to the manufacturer’s instructions [29].

qRT-PCR analyses were performed using the SensiFast Probe No-ROX One-Step Kit, supplemented with SYBR Green dye before the amplification process. Reactions were carried out on a QuantStudio 5 real-time PCR system [15,30]. The thermal protocol began with a reverse transcription step at 42 °C for 5 min, followed by 40 amplification cycles. Each cycle included a denaturation phase at 95 °C for 5 s, an annealing step at 58 °C for 15 s, and an extension phase at 72 °C for 15 s. Fluorescence signals were measured at 520 nm during the extension step [15,30]. Gene expression levels were normalized using *GAPDH* as the internal control. Primer sequences are shown in Table 2.

### 2.6. MiRs Extraction, RT and qPCR

MiRs from synovial tissue-derived MSC-like cells stimulated with poly I:C (10 µg/mL), IL-1β, TFN-α, IFN-γ, TGF-β1 and PDGF-BB (20 ng/mL) in 6-well plate using the miRNeasy Serum/Plasma Advanced Kit (Qiagen, Hilden, Germany, Ref. 217204). MiRs from synovial tissue-derived MSC-like cells transfected with miR-146a-5p or miR-221-3p (100 nM) and/or stimulated with TNF-α were directly extracted from harvested cell culture in 12-well plate using the same extraction kit. Before miR extraction, 250 µL of NaCl (0.09%) were added in each well, collected and kept at −80 °C until use.

MiRs from plasma of patients with RA or HC were also extracted using the same extraction kit.

MiRs were isolated from 200 µL of plasma, with a synthetic spike-in control added at 1.6 × 10^8^ copies/µL at the start of extraction. Reverse transcription was conducted using the miScript II RT kit in a total reaction volume of 20 µL, comprising 5 µL of extracted RNA and 15 µL of enzyme mix, as previously described [15,30]. qPCR was performed using the miScript SYBR Green PCR kit (Qiagen, Ref. 218075) in a total volume of 5 µL per reaction, consisting of 1 µL of cDNA, 3 µL of master mix, and 1 µL of primer solution (final primer solution: 1.15 µM). Amplification was carried out on a QuantStudio 5 thermocycler [15,30].

Relative miR expression was calculated using Ce-39 (Qiagen ref. MS00019789). Samples were analyzed from five independent experiments. Primers used for miR analysis are shown in Table 3.

### 2.7. Enzyme-Linked Immunosorbent Assay (ELISA) for CXCL8, CCL-2, IL1-β, IL-6 and VEGF

Commercial ELISA kits for CCL2 (Peprotech, Rocky Hill, CT, USA, cat. No. 900-T31), CXCL8 (Peprotech, Rocky Hill, CT, USA, cat. No. 900-K18), IL1-β (Peprotech, Rocky Hill, CT, USA, cat.no 900-K95) were used to measure the concentrations of CCL2, CXCL8 and IL1-β in plasma of RA patients or HC patients according to the manufacturer’s instructions. CCL2, CXCL8, IL-1β and VEGF (Peprotech, Rocky Hill, CT, USA, cat. No. 900-K10) and IL-6 (Peprotech, Rocky Hill, CT, USA, cat. No. 900-T16) protein expressions were determined in synovial tissue-derived MSC-like cells transfected with miR-146a-5p or miR-221-3p or non-transfected and stimulated or not with TNF-α. Samples were analyzed from five independent experiments.

### 2.8. Statistical Analysis

Statistical analysis was performed using GraphPad Prism 8 software.

For plasma samples, the distribution of miR expression levels and any additional plasma-derived variables was evaluated for normality using the Shapiro–Wilk test. Since the data did not follow a normal distribution (*p* < 0.005), comparisons between two independent groups were carried out using the non-parametric Wilcoxon–Mann–Whitney test [31]. Reported values are presented median [IQR].

For cell culture experiments, comparisons were performed between multiple experimental groups. Data were first assessed for normality with the Shapiro–Wilk method. For datasets that met normality assumptions, differences between groups were evaluated by one-way ANOVA, followed by Bonferroni post hoc correction for multiple comparisons. When normality was not satisfied, the non-parametric Kruskal–Wallis test followed by Dunn’s post hoc test was applied [32]. Results are presented as means ± SEM.

## 3. Results

### 3.1. Altered Expression of miRs in RA

**Figure 1:**
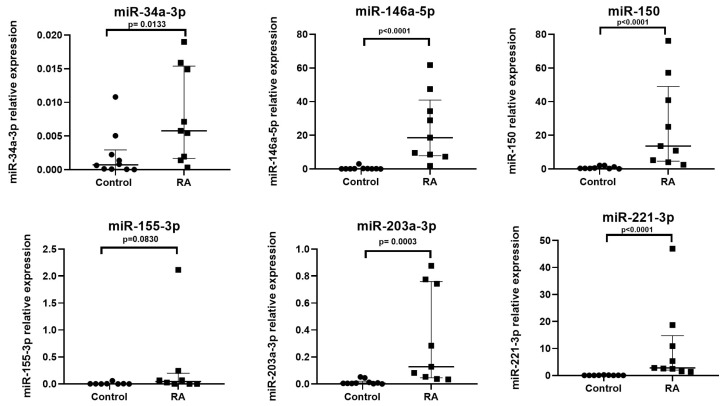
Increased expression of miRs in RA. Plasma samples from RA patients (*n* = 9) and HC (*n* = 10) were collected and miR expression was determined by qPCR. Reported values are presented median [IQR]. Relative expression of miRNAs was determined using the ΔΔCt method and normalized to the spike-in control Ce-39. Expression levels are shown relative to the control group. Statistical comparisons between groups were performed using Wilcoxon–Mann–Whitney test. All experiments were performed in duplicate (technical duplicate) and from a single experiment.

First, we determined the expression of miRs in RA patient plasma compared to healthy control (HC). MiR-34a-3p (up to 3-fold), miR-146a-5p (up to 89-fold), miR-150 (up to 27-fold), miR-203a-3p (up to 21-fold) and miR-221-3p (up to 72-fold) expressions were upregulated in the plasma of RA patients as compared to HC. There was no significant difference for miR-155-3p expression between the two groups (Figure 1).

### 3.2. Increased Expression of Pro-Inflammatory Mediators in RA

**Figure 2:**
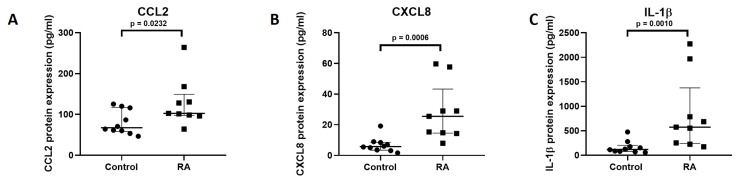
Increased expression of pro-inflammatory mediators in RA. Protein levels of CCL2 (**A**), CXCL8 (**B**) and IL-1β (**C**) were determined by ELISA test in plasma of RA patients (*n* = 9) and HC (*n* = 10). Reported values are presented median [IQR]. Statistical comparisons between groups were performed using Wilcoxon–Mann–Whitney test.

Then, we determined the protein expression of two chemokines (CCL2 and CXCL8) and the pro-inflammatory cytokine IL-1β in RA vs HC. Protein levels of CCL2 (2-fold) (Figure 2A), CXCL8 (4-fold) (Figure 2B) and IL-1β (5-fold) (Figure 2C) were significantly increased in RA patients as compared to HC.

### 3.3. Expression of MSC Markers in Our In Vitro Model

**Figure 3:**
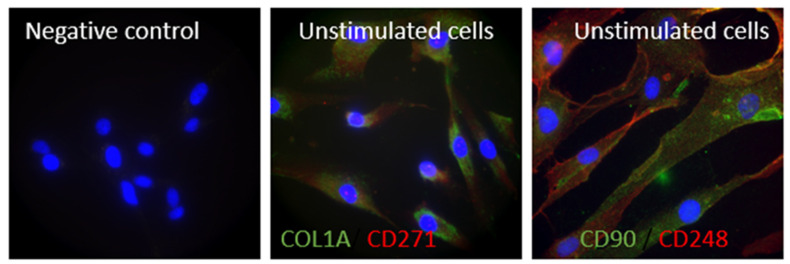
Synovial tissue-derived MSC-like cells expressed markers of MSC. Synovial tissue-derived MSC-like cells were probed with primary antibody mouse anti-CD271 (Biolegend ref. 839701, Amsterdam, The Netherlands) and rabbit anti-collagen 1A (mouse IgG anti-human collagen I M38 clone) or with mouse anti-CD90 (Cell signalling ref.13801S, Danvers, MA, USA) and rabbit anti-CD248 (cell signalling ref.47948S, Danvers, MA, USA). Nuclei were stained by DAPI (Sigma ref. D952, Lyon, France). Alexa Fluor 594-conjugated donkey anti-rabbit antibody (red) (Invitrogen ref. 121207, Thermo Fisher Scientific, Waltham, MA, USA) and Alexa488-conjugated donkey anti-mouse antibody (green) (Invitrogen ref. A21202) were used as secondary antibodies.

We showed by immunofluorescence that synovial tissue-derived MSC-like cells expressed markers of MSC such as CD271, CD90 and CD248 (Figure 3).

### 3.4. Modified Expression of miR-146a-5p and miR-221-3p in Response to Pro-Inflammatory Mediators

**Figure 4:**
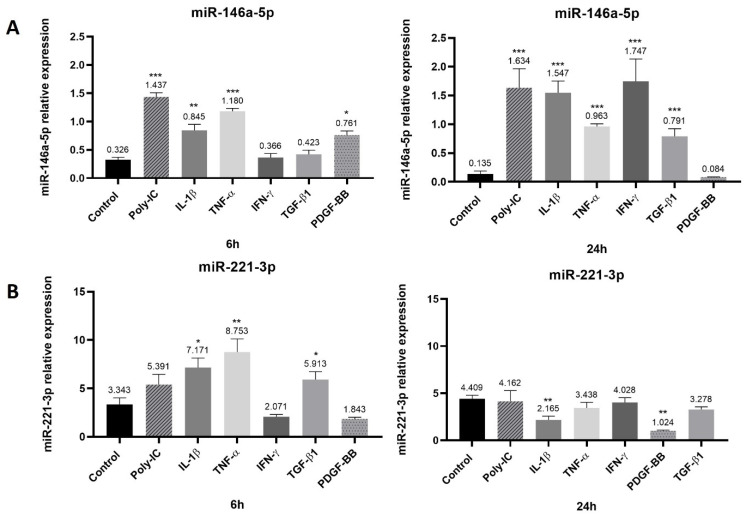
Modified expression of miR-146a-5p and miR-221-3p in response to pro-inflammatory cytokines and growth factors. Human synovial tissue-derived MSC-like cells were stimulated with poly I:C (10 µg/mL), IL-1β, TNF-α, IFN-γ, TGF-β1 and PDGF-BB (20 ng/mL) during 6 h and 24 h. qPCR was performed to determine the expression of miR-146a-5p (**A**) and miR-221-3p (**B**). Reported values are means ± SEM and *p*-value was calculated using ANOVA Bonferroni test: *: *p* < 0.05, **: *p* < 0.01 as compared to control, ***: *p* < 0.001. Experiments were performed in three independent experiments (*n* = 3).

The expression of several miRs was determined in synovial tissue-derived MSC-like cells stimulated with pro-inflammatory mediators or growth factors. MiR-146a-5p was upregulated in response to stimulation with poly I:C as compared to control (untreated cells) at 6 (up to 4-fold) and 24 h (up to 12-fold). The pro-inflammatory cytokines IL-1β and TNF-α significantly increased miR-146a-5p expression at 6 h (up to 3-fold and 4-fold, respectively) and 24 h (up to 11-fold and 7-fold, respectively). IFN-γ increased miR-146a-5p expression at 24 h (13-fold). TGF-β1 (transforming growth factor beta 1) significantly increased miR-146a-5p expression at 24 h up to 6-fold and PDGF-BB (platelet-derived growth factor BB) increased miR-146a-5p at 6 h (2-fold) (Figure 4A). MiR-221-3p expression was significantly increased in response to stimulation with IL-1β (2-fold) and TNF-α (3-fold) at 6 h. However, at 24 h, IL-1β significantly decreased miR-221-3p expression. TGF-β1 also increased miR-221-3p at 6 h (2-fold) and PDGF-BB decreased its expression at 24 h (Figure 4B). All of the treatments had no effect on miR-155-3p, miR-203a-3p, miR-34a-3p and miR-150 expression at 6 h and 24 h. Moreover, miR-203a-3p, miR-34a-3p and miR-150 were weakly expressed in synovial tissue-derived MSC-like cells (Figure S1).

### 3.5. Confirmation of Synovial Tissue-Derived MSC-like Cells Transfection by miR-146a-5p and miR-221-3p

**Figure 5:**
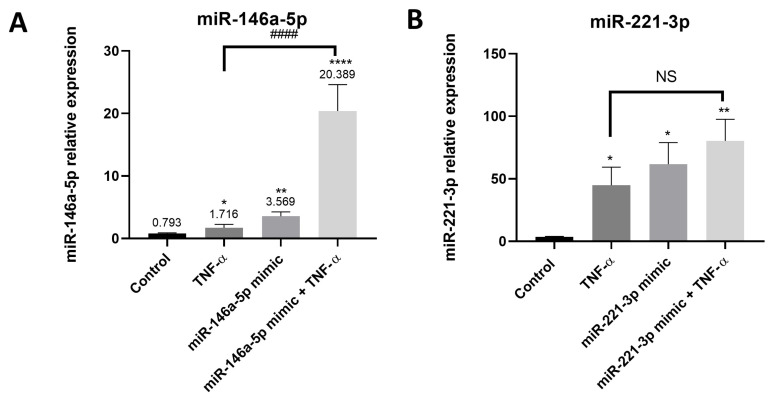
Confirmation of synovial tissue-derived MSC-like cells transfection by miR-146a-5p mimic and miR-221-3p mimic. Synovial tissue-derived-MSC-like cells were transfected with miR-146a-5p mimic or miR-221-3p mimic (100 nM) using lipofectamine 3000 or non-transfected (+lipofectamine) during 48 h. Then, transfected cells were stimulated with TNF-α during 6 h, non-transfected cells were not stimulated (control) or stimulated with TNF-α during 6 h. Expression of (**A**) miR-146a-5p and (**B**) miR-221-3p was determined by qPCR. Reported values are mean ± SEM and *p*-value was calculated using Kruskal–Wallis test followed by Dunn’s post hoc test: *: *p* < 0.05, **: *p* < 0.01, ****: *p* < 0.0001 as compared to control (non-transfected cells); ####: *p* < 0.0001 as compared to TNF-α. Experiments were performed in five independent experiments (*n* = 5). NS: Not significant.

MiR-146a-5p and miR-221-3p were selected for the following experiments. We confirmed that the transfection of synovial tissue-derived MSC-like cells by miR-146a-5p mimic increased miR-146a-5p expression (up to 2-fold). When cells were transfected with miR-146a-5p mimic and then stimulated with TNF-α for 6 h, miR-146a-5p expression was significantly upregulated as compared to control (non-transfected cells) (2-fold) and as compared to cells stimulated with TNF-α alone (15-fold) (Figure 5A). Transfection of synovial tissue-derived MSC-like cells with miR-221-3p significantly upregulated the expression of its miR (18-fold). Transfection of cells with miR-221-3p and stimulation with TNF-α also increased miR-221-3p as compared to control (non-transfected cells) (23-fold) (Figure 5B).

### 3.6. Increased Expression of Chemokines in Synovial Tissue-Derived MSC-like Cells Transfected with miR-146a-5p and Stimulated with TNF-α

**Figure 6:**
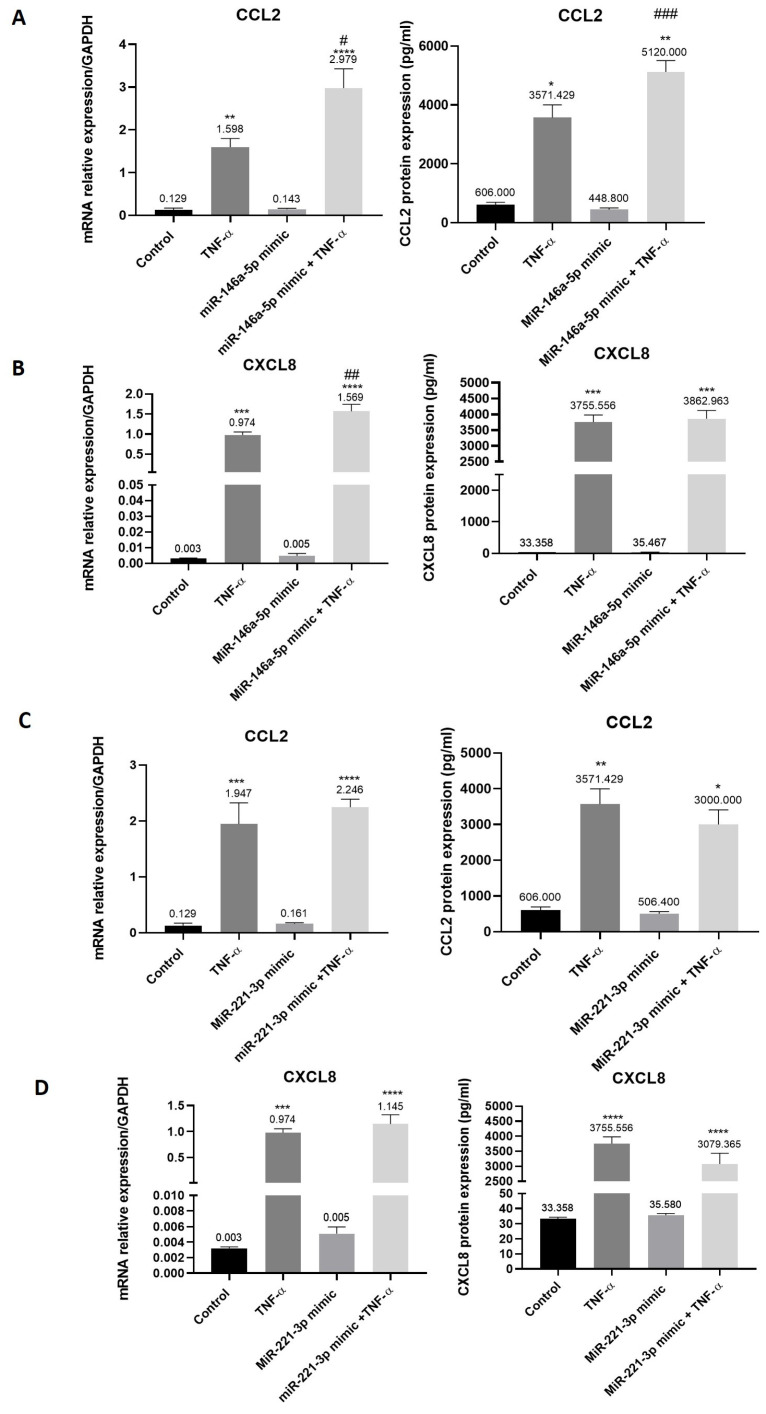
Increased expression of chemokines in synovial tissue-derived MSC-like cells transfected with miR-146a-5p and stimulated with TNF-α. Synovial tissue-derived MSC-like cells were transfected with miR-146a-5p mimic or miR-221-3p mimic and then stimulated with TNF-α during 6 h as previously described. mRNA and protein expression of (**A**) CCL2 and (**B**) CXCL8 in miR-146a-5p transfected cells were determined by qRT-PCR and ELISA tests. (**C**) CCL2 and (**D**) CXCL8 mRNA and protein expression were determined in miR-221-3p transfected cells. Reported values are mean ± SEM. Data with a normal distribution were analyzed using one-way ANOVA followed by Bonferroni’s post hoc test ((**A**,**B**): *CCL2* and *CXCL8* mRNA expression; (**C**): *CCL2* mRNA expression; (**D**): CXCL8 mRNA and protein expression, whereas non-normally distributed parameters were analyzed using the Kruskal–Wallis test followed by Dunn’s post hoc test ((**A**,**B**), CCL2 and CXCL8 protein expression; (**C**): CCL2 protein expression). Significance is indicated as follows: *: *p* < 0.05, **: *p* < 0.01, ***: *p* < 0.001, ****: *p* < 0.0001 versus control (non-transfected cells); #: *p* < 0.05, ##: *p* < 0.01, ###: *p* < 0.001 versus TNF-α-treated cells. Experiments were performed in five independent replicates (*n* = 5).

Transfection of synovial tissue-derived MSC-like cells with miR-146a-5p had no effect on *CCL2* and *CXCL8* mRNA and protein expression as compared to non-transfected cells (Figure 6A,B). The same results were observed for cells transfected with miR-221-3p (Figure 6C,D). Stimulation of miR-146a-5p transfected cells with TNF-α increased CCL2 (up to 23-fold) and CXCL8 (up to 448-fold) mRNA and protein expression (up to 8-fold and up to 116-fold, respectively) as compared to non-transfected cells (control) (Figure 6A,B). The same results were observed in miR-221-3p transfected cells (Figure 6C,D). Interestingly, when miR-146a-5p transfected cells were stimulated with TNF-α, mRNA and protein expression of CCL2 (up to 1.9- and 1.4-fold, respectively) significantly increased as compared to non-transfected cells stimulated with TNF-α (Figure 6A). *CXCL8* mRNA expression was also increased in TNF-α miR-146a-5p transfected cells as compared to non-transfected cells stimulated with TNF-α (1.6-fold) (Figure 6B).

### 3.7. Increased Expression of Cytokines in Synovial Tissue-Derived MSC-like Cells Transfected with miR-221-3p and Stimulated with TNF-α

**Figure 7:**
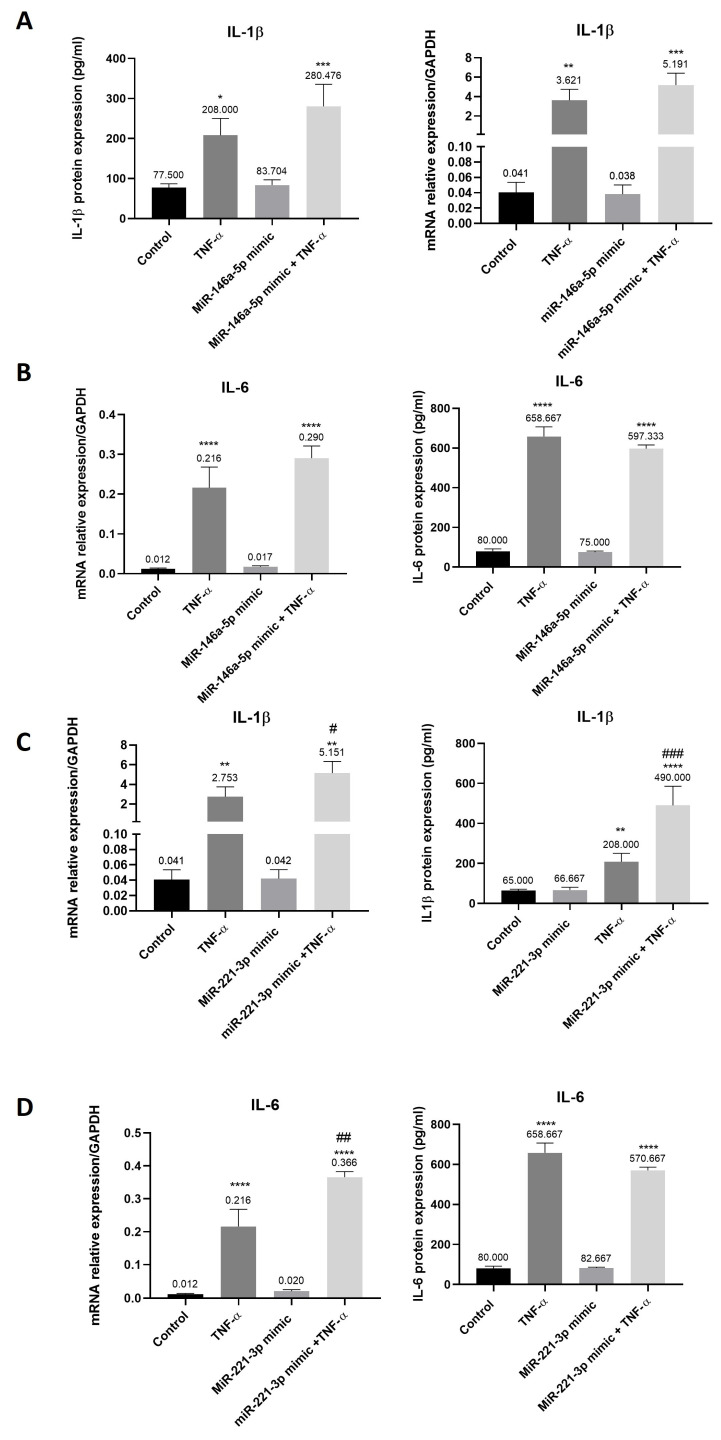
Increased expression of pro-inflammatory cytokines in synovial tissue-derived MSC-like cells transfected with miR-221-3p and stimulated with TNF-α. Synovial tissue-derived MSC-like cells were transfected with miR-146a-5p mimic or miR-221-3p mimic and stimulated with TNF-α as previously described. mRNA and protein expression of (**A**) IL-1β and (**B**) IL-6 in miR-146a-5p transfected cells were determined by qRT-PCR and ELISA tests. (**C**) IL-1β and (**D**) IL-6 mRNA and protein expression were also determined in miR-221-3p transfected cells. Reported values are mean ± SEM. Data with a normal distribution were analyzed using one-way ANOVA followed by Bonferroni’s post hoc test ((**A**,**B**): IL-1β protein expression, IL-6 mRNA and protein expression; (**C**,**D**): IL-1β and IL-6 mRNA and protein expression), whereas non-normally distributed parameters were analyzed using the Kruskal–Wallis test followed by Dunn’s post hoc test ((**A**,**B**), IL-1β mRNA expression). Significance is indicated as follows: *: *p* < 0.05, **: *p* < 0.01, ***: *p* < 0.001, ****: *p* < 0.0001 as compared to control (non-transfected cells); #: *p* < 0.05, ##: *p* < 0.01, ###: *p* < 0.001 as compared to TNF-α. Experiments were performed in five independent experiments (*n* = 5).

MiR-146a-5p had no effect on IL-1β and IL-6 mRNA and protein expression (Figure 7A,B). The same results were observed for miR-221-3p (Figure 7C,D). When miR-146a-5p transfected cells were stimulated with TNF-α, mRNA and protein expression of IL-1β (up to 128-fold and up to 4-fold, respectively) and IL-6 (up to 25-fold and up to 7-fold, respectively) were significantly increased as compared to non-transfected cells (Figure 7A,B). The same results were observed for miR-221-3p transfected cells (Figure 7C,D). Interestingly, the stimulation of miR-221-3p transfected cells with TNF-α significantly upregulated mRNA and protein expression of IL-1β (up to 2-fold) as compared to non-transfected cells stimulated with TNF-α (Figure 7C). *IL-6* mRNA expression was also increased in miR-221-3p transfected cells stimulated with TNF-α (2-fold) as compared to non-transfected cells stimulated with TNF-α (Figure 7D).

## 4. Discussion

First, synovial biopsies were performed on small joints of RA patients; therefore, the amounts of synovial tissues were not sufficient for these analyses. Therefore, we used commercial synovial cells (primary culture). We validated that these cells are representative of RA synovial tissue-derived cells in vitro. Indeed, we showed that stimulation of synovial tissue-derived MSC-like cells with Poly I:C (known to mimic viral infection) or pro-inflammatory mediators expressed in RA (IL-1β and TNF-α) can induce inflammation, expression of *MMPs*, *VEGF* and *RANKL* such as described in RA (Figure S5). Together, these results indicate that our model is a representative model of RA in vitro. Moreover, we confirmed by immunofluorescence that our cells expressed marker of MSC such as CD90, CD248 and CD271. The in vitro model used in this study relies on TNF-α stimulation of synovial tissue-derived MSC, a widely accepted approach to reproduce key features of the inflammatory microenvironment observed in RA. Although additional phenotypic characterization following stimulation—particularly regarding the expression of markers such as CD90, CD271, and CD248—would provide further insight into MSC-like cell activation status, such analyses could not be performed within the scope of the present study. Nevertheless, previous studies have consistently shown that TNF-α is able to modulate MSC phenotype, including surface marker expression and immunomodulatory properties, and can drive these cells toward a more activated or pro-inflammatory profile depending on the context [33,34,35].

The characteristics of RA patients and HC should also be considered when interpreting circulating biomarker levels. In our cohort, RA patients and HC were comparable in terms of age though sex distribution differed. Eight out of nine RA patients were on DMARDs whereas HC were treatment-naïve. Disease activity parameters were recorded when available, with heterogeneity observed among RA patients. In addition, 78% of RA patients were ACPA-positive. ACPA positivity is associated with a more pronounced autoimmune and inflammatory profile in RA and may influence circulating miR expression. Indeed, ACPA-positive RA has been linked to enhanced immune activation and cytokine production [36], which may influence the expression of inflammation-related miRs. Several miRs involved in inflammatory pathways, including miR-146a-5p and miR-221-3p, have been reported to be dysregulated in RA and associated with immune cell activation, cytokine signalling and synovial inflammation [37,38]. DMARD therapy can influence systemic inflammatory status and circulating miR expression. Indeed, anti-rheumatic treatments targeting inflammatory pathways may modulate cytokine, chemokine and miR expression levels. Therefore, the differential expression of circulating biomarkers observed in our study should be interpreted in the context of systemic inflammation and clinical heterogeneity within the cohort.

Recent studies have suggested that RA is a multifactorial disease involving complex interactions between synovial cells, immune cells, cytokines and other inflammatory mediators [39]. In this context, miRs may participate in the epigenetic regulation of inflammatory and tissue-destructive processes.

As miRs might participate to inflammation in RA, we determined the expression of six miRs (miR-146a-5p, miR34a-3p, miR-150, miR-203a-3p, miR-221-3, miR-155-3p) in the plasma of RA patients vs HC. These micro-RNAs were chosen based on previous evidence showing their altered expression in rheumatoid arthritis.

As described in the literature, we found that miR-146a-5p expression was upregulated in plasma of RA patients as compared to HC [40]. We showed for the first time that miR-34a-3p was upregulated in RA as compared to HC. Renman et al. described that there was no significant difference between miR-34a-3p expression in RA vs HC [41]. Moreover, we demonstrated that miR-150 was upregulated in the plasma of RA patient’s vs HC. This miR was described to be upregulated in PBMC [42] and whole blood samples of RA patients [43]. MiR-203a-3p was also upregulated in the plasma of RA patients vs HC and has been reported to be upregulated in synovial tissue-derived MSC-like cells of RA patients [44]. MiR-221-3p was upregulated in the plasma of RA patient’s vs HC. MiR-221-3p is reported to increase in synovial tissue and fluid of RA patients [45]. We can note miR-146a-5p, miR-150 and miR-221-3p were highly expressed in the plasma of RA patients. These results suggest a potential role of these circulating miRs in systemic inflammatory signalling. It is already known that chemokines and pro-inflammatory cytokines participate to RA pathogenesis [46,47]. Therefore, we determined the protein expression of two chemokines, CCL2 and CXCL8 and the pro-inflammatory cytokine IL-1β in plasma samples of RA patients. As expected, these pro-inflammatory mediators were upregulated in the plasma of RA patients as compared to HC.

As miRs were reported to be differentially expressed in synovial tissue-derived MSC-like cells of RA patients [1], we determined if miR-146a-5p, miR-155-3p, miR-203a-3p, miR-221-3p, miR-150 and miR-34a-3p were expressed and regulated in an in vitro model of RA. Therefore, in order to mimic the pathogenic setting of RA, synovial tissue-derived MSC-like cells were stimulated with poly I:C (to mimic viral infection), IL-1β, TNF-α, IFN-γ, TGF-β1 and PDGF-BB and the expression of miRs was determined by RT-PCR. We found that miR-146a-3p expression was increased in cells stimulated by IL-1β. This result was in accordance with a previous study describing an increased expression of miR-146a-5p in RA synovial tissue-derived MSC-like cells stimulated with IL-1β [19]. Moreover, we showed that poly I:C and TNF-α also induced miR-146a expression. However, Stanczyk et al. showed that poly I:c and TNF-α failed to increase miR-146a expression in RA synovial tissue-derived MSC-like cells [19]. The growth factors TGF-β1 and PDGF-BB also increased miR-146a-5p expression (at 24 h and 6 h, respectively). Increased expression of miR-221-3p was also observed in cells stimulated with IL-1β, TNF-α and TGF-β1 at 6 h. MiR-221 is upregulated in response to LPS [24] and TNF-α [48]. These results showed that in the presence of pro-inflammatory mediators, miR-146a-5p and miR-221-3p were upregulated in synovial tissue-derived MSC-like cells suggesting that the pro-inflammatory environment may influence the expression of these two miRs. Expression of miR-34a-3p, miR-150, miR-155-3p and miR-203a-3p was also determined in our in vitro model. However, these miRs were weakly expressed (Figure S1). Therefore, we selected miR-146a-5p and miR-221-3p for further experiments.

In RA, activated synovial tissue-derived MSC-like cells expressed pro-inflammatory mediators, pro-angiogenic factors, MMPs and pro-osteoclastic factors [1,4,7]. The acquisition of this pathogenic phenotype might be due to an epigenetic reprogramming of these cells. Therefore, in order to determine the effect of miR-146a-5p and miR-221-3p on pro-inflammatory, pro-angiogenic, pro-osteoclastic and enzymes involved in cartilage degradation, we transfected miR-146a-5p and miR-221-3p in synovial tissue-derived MSC-like cells stimulated with TNF-α (to mimic clinical effects of RA) or unstimulated. We confirm cell transfection by qRT-PCR. We showed that CCL2 and CXCL8 protein and/or mRNA expression was upregulated in miR-146a-5p transfected cells as compared to TNF-α-stimulated cells. Moreover, no differences were observed concerning IL-1β and IL-6 expressions.

These results indicated that transfection of synovial tissue-derived MSC-like cells by miR-146a-5p altered the response of these cells to TNF-α, which was manifested by the upregulation of CCL2 and CXCL8 expression. These results suggest that miR-146a-5p may potentiate chemokine-mediated immune cell recruitment in the RA synovial microenvironment. MiR-146a has been reported to regulate inflammatory phenotypes of immune cells, including T regulatory cells via *STAT1* signalling [49]. However, other studies have reported anti-inflammatory effects of miR-146a through inhibition of the *TLR4/NF-κB* signalling pathway [23], suggesting a context-dependent and cell-type specific role of this miR in RA pathogenesis.

Next, we showed that overexpression of miR-146a-5p in synovial tissue-derived MSC-like cells increased *VEGF* mRNA and protein expression, suggesting the implication of this miR in angiogenesis (Figure S2). We also showed that miR-146a-5p has no effect on *MMP-1* and *MMP-3* (Figure S3). Nevertheless, another study described that miR-146a decreased MMP-3 expression [23]. We also found that overexpression of miR-146a-5p has no effect on *RANKL* expression (Figure S4). The role of miR-146a in RA has been widely investigated and remains controversial, as both pro- and anti-inflammatory effects have been reports depending on the cellular context. In the present study, we provide additional insight into this issue by demonstrating that miR-146a-5p enhances the inflammatory response of synovial tissue-derived MSC-like cells to TNF-α, notably through increased expression of CCL2 and CXCL8. Our results therefore suggest that, in synovial MSC, miR-146a-5p may contribute to the amplification of inflammatory responses in the RA microenvironment.

Next, in synovial tissue-derived MSC-like cells transfected with miR-221-3p and stimulated with TNF-α, expression of IL-1β (mRNA and protein) and *IL-6* (mRNA) was increased as compared to cells stimulated with TNF-α. These results suggest that overexpression of miR-221-3pp favoured the expression of pro-inflammatory cytokines by synovial tissue-derived MSC-like cells in response of TNF-α. Therefore, miR-221-3p might favour inflammation in RA. Overexpression of miR-221-3p has no effect on chemokines expression in synovial tissue-derived MSC-like cells stimulated with TNFα. MiR-221 was described to favoured inflammation in RA. Indeed, inhibition of miR-221 decreased the expression of *TNF-α*, *IL-6* and *IL-1β* in synovial tissue-derived MSC-like cells stimulated with LPS. Moreover, miR-221 has also been described to promote synovial tissue-derived MSC-like cell proliferation [24]. Effectively, inhibition of miR-221 decreased *VEGF* expression. However, herein, we showed that miR-221-3p has no effect on *VEGF* mRNA expression (Figure S2). We also showed that miR-221-3p has no effect on *MMPs* and *RANKL* expression (Figure S3 and Figure S4, respectively).

Overall, these results support the hypothesis that miR-146a-5p and miR-221-3p act as functional regulators of the inflammatory synovial microenvironment. Rather than acting as simple biomarkers, these miRs may participate in the regulation of multiple pathogenic processes in RA, consistent with the multifactorial nature of the disease.

The strength of our study is that we determined the expression of miRs in plasma and in synovial tissue-derived MSC. We showed that miR-146a-5p and miR-221-3p expressed by synovial tissue-derived MSC-like cells are also present in plasma. These results suggest that miR-146a-5p and miR-221-3p are circulating miRs. Furthermore, we used an in vitro model representative of the pathological setting of RA to study the involvement of miRs in RA pathogenesis. We also confirmed that miR-146a-5p and miR-221-3p might be pro-inflammatory miRs in this context.

Although we identified differential expression of circulating and cellular miRs associated with inflammatory mediators, these findings are not sufficient to definitively establish miR-146a-5p and miR-221-3p as reliable diagnostic or prognostic biomarkers for rheumatoid arthritis. Validation in larger, independent cohorts with well-characterized clinical and biological characteristics will be necessary to confirm the clinical relevance and robustness of these miRs.

One of the limitations of the study is that we used miRs mimics to transfect cells. The use of miR inhibitors (antago-miR) could have been useful in order to confirm our results. Regulation and function of miR-146a-5p and miR-221-3p will need to be assessed ex vivo in synovial tissue of RA patients. Moreover, complementary loss-of-function experiments using miRs inhibitors (antago-miR) would be useful to further confirm the functional role of these miRs in the inflammatory response of these cells. In addition, the identification of direct molecular targets of miR-146a-5p and miR-221-3p in synovial tissue-derived MSC-like cells will require additional mechanistic studies such as luciferase reporter assays. These experiments will be necessary to better define the molecular pathways through which these miRs regulate inflammation in RA.

Another limitation of this study is the relatively small size of the patient cohort, which may limit the statistical power of the analyses and the generalizability of our findings. In addition, potential cohort heterogeneity, including variability in disease duration, treatment regimens, and disease activity status among RA patients, may have influenced miR and cytokine expression levels.

## 5. Conclusions

**Figure 8:**
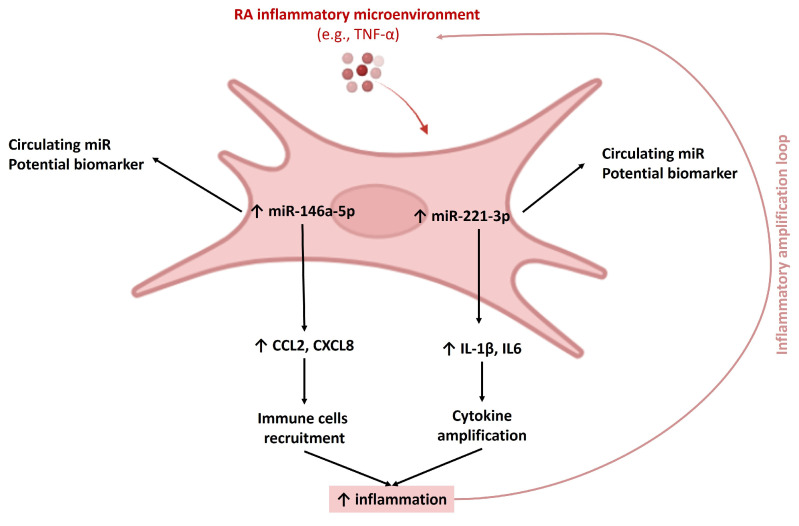
Overexpression of miR-146a-5p and miR-221-3p might favour inflammation in RA.

In conclusion, our results demonstrate that pro-inflammatory mediators induced the expression of miR-146a-5p and miR-221-3p in synovial tissue-derived MSC. Functionally, compared with TNF-α-stimulated cells, overexpression of miR-146a-5p increased CCL2 mRNA and protein expression and *CXCL8* mRNA expression, while overexpression of miR-221-3p increased IL-1β mRNA and protein expression and *IL-6* mRNA expression. These findings suggest that miR-146a-5p and miR-221-3p may contribute to the amplification of inflammatory responses within the synovial microenvironment in RA, and importantly, these miRs are also detectable as circulating miRs in plasma, supporting their potential role as systemic mediators or inflammation (Figure 8). Together, these results highlight both cellular and circulating miRs as potential players in RA pathogenesis, suggesting that they may represent promising candidate biomarkers of diseases activity and potential therapeutic targets, although further validation is required before clinical translation.

Further studies are required to better elucidate the molecular mechanisms underlying these effects, particularly through the identification of the downstream targets of these miRs and functional validation in ex vivo synovial tissues from RA patients. Future work should also include validation in larger patient cohorts to confirm the clinical relevance of these findings. Loss-of-function approaches, such as antago-miR, together with mechanistic assays such as luciferase reporter analyses, will be necessary to better characterize the signalling pathways regulated by these miRs in synovial inflammation.

Our study indicates that miR-146a-5p and miR-221-3p, both intracellularly in synovial tissue-derived MSC-like cells and as circulating miRs, may participate in RA pathogenesis, but their precise clinical and functional significance remains to be further confirmed in larger populations.

## Figures and Tables

**Table 1:** Demographic and clinical characteristics of RA and HC patients.

RA patients	
Characteristics	Number of patient *n* = 9 (100%)
Male sex, *n* (%)	4 (44%)
Age years, median (IQR)	56 (53–62)
Duration of the diseases years, median (IQR)	12 (6–15)
ACPA-positive, *n* (%)	7 (78%)
DMARDs, *n* (%)	8 (89%)
DAS 28, median (IQR)	2.9 (2.5–4.0)
C-reactive protein (mg/L), median (IQR)	4 (2–25)
Healthy Control	
Characteristics	Number of patient *n* = 10 (100%)
Male sex, *n* (%)	7 (70%)
Age years, median (IQR)	57 (38–73)
Treatments, *n* (%)	0 (0%)

**Table 2:** Sequences of primers used for qRT-PCR analyses.

Target Genes	Forward (5′-3′)	Reverse (3′-5′)
*GAPDH*	TGCGTCGCCAGCCGAG	AGTTAAAAGCAGCCCTGGTGA
*CCL2*	CTGCTCATAGCAGCCACCTT	CTTGAAGATCACAGCTTCTTTGGG
*CXCL8*	CAGAGACAGCAGAGCACACA	GGCAAAACTGCACCTTCACA
*IL-1β*	TTGCTCAAGTGTCTGAAGCAG	GGTGGTCGGAGATTCGTAGC
*IL-6*	TACATCCTCGACGGCATCTC	ACCAGGCAAGTCTCCTCATTG
*MMP-1*	TTTGTCAGGGGAGATCATCGG	TCCAAGAGAATGGCCGAGTT
*MMP-3*	TCAGTCCCTCTATGGACCTCCC	GGTTCAAGCTTCCTGAGGGAT
*VEGF*	AGGCCAGCACATAGGAGAGA	ACGCGAGTCTGTGTTTTTGC

**Table 3:** Sequences of miRs primers used.

Target miR	Forward (5′-3′)
hsa-miR-34a-3p	CGCAGCAATCAGCAAGT
hsa-miR-146a-5p	GCAGTGAGAACTGAATTCCATG
hsa-miR-150	CTCCCAACCCTTGTACCA
hsa-miR-155-3p	CGCAGCTCCTACATATTAGCA
hsa-miR-203a-3p	CGCAGGTGAAATGTTTAGGA
hsa-miR-221-3p	GCAGAGCTACATTGTCTGCT

## Data Availability

The datasets used and/or analyzed during the current study available from the corresponding author on reasonable request.

## Supplementary Materials

The following supporting information can be downloaded at: https://www.mdpi.com/article/10.3390/cells15080691/s1, Figure S1: Pro-inflammatory mediators and growth factors had no effect on miR-155-3p, miR-203a-3p, miR-34a-3p and miR-150 expression; Figure S2: miR-146a-5p upregulated VEGF expression; Figure S3: MiR-146a-5p and miR-221-3p did not affect the expression of MMPs in synovial tissue-derived MSC-like cells stimulated with TNF-α; Figure S4: MiR-146a-5p and miR-221-3p did not affect RANKL mRNA expression in synovial tissue-derived MSC. Figure S5: Expression of pro-inflammatory mediators, MMPs, VEGF and RANL in response to pro-inflammatory stimuli.

## Abbreviations

DAMPs	Danger Associated Molecular Patterns
CCL2	Chemokine ligand 2
CXCL8	CXC motif chemokine ligand 8
HC	Healthy controls
MiRs	Micro-RNAs
MMPs	Metalloproteinases
MSC	Mesenchymal Stem Cells
PAMPs	Pathogen Associated Molecular Patterns
RA	Rheumatoid Arthritis
RANKL	Receptor Activator of NF-κB Ligand
VEGF	Vascular Endothelial Growth Factor

## References

[1] Bottini N., Firestein G.S. (2013). Duality of Fibroblast-like Synoviocytes in RA: Passive Responders and Imprinted Aggressors. Nat. Rev. Rheumatol..

[2] Nygaard G., Firestein G.S. (2020). Restoring Synovial Homeostasis in Rheumatoid Arthritis by Targeting Fibroblast-like Synoviocytes. Nat. Rev. Rheumatol..

[3] Smolen J.S., Aletaha D., McInnes I.B. (2016). Rheumatoid Arthritis. Lancet.

[4] McInnes I.B., Schett G. (2011). The Pathogenesis of Rheumatoid Arthritis. N. Engl. J. Med..

[5] Georganas C., Liu H., Perlman H., Hoffmann A., Thimmapaya B., Pope R.M. (2000). Regulation of IL-6 and IL-8 Expression in Rheumatoid Arthritis Synovial Fibroblasts: The Dominant Role for NF-Kappa B but Not C/EBP Beta or c-Jun. J. Immunol..

[6] Sabeh F., Fox D., Weiss S.J. (2010). Membrane-Type I Matrix Metalloproteinase-Dependent Regulation of Rheumatoid Arthritis Synoviocyte Function. J. Immunol..

[7] Yoshitomi H. (2019). Regulation of Immune Responses and Chronic Inflammation by Fibroblast-Like Synoviocytes. Front. Immunol..

[8] Phuklia W., Kasisith J., Modhiran N., Rodpai E., Thannagith M., Thongsakulprasert T., Smith D.R., Ubol S. (2013). Osteoclastogenesis Induced by CHIKV-Infected Fibroblast-like Synoviocytes: A Possible Interplay between Synoviocytes and Monocytes/Macrophages in CHIKV-Induced Arthralgia/Arthritis. Virus Res..

[9] Ritchlin C. (2000). Fibroblast Biology: Effector Signals Released by the Synovial Fibroblast in Arthritis. Arthritis Res..

[10] Jackson J.R., Minton J.A., Ho M.L., Wei N., Winkler J.D. (1997). Expression of Vascular Endothelial Growth Factor in Synovial Fibroblasts Is Induced by Hypoxia and Interleukin 1beta. J. Rheumatol..

[11] Paleolog E.M., Young S., Stark A.C., McCloskey R.V., Feldmann M., Maini R.N. (1998). Modulation of Angiogenic Vascular Endothelial Growth Factor by Tumor Necrosis Factor Alpha and Interleukin-1 in Rheumatoid Arthritis. Arthritis Rheum..

[12] Lebeau G., Ah-Pine F., Daniel M., Bedoui Y., Vagner D., Frumence E., Gasque P. (2022). Perivascular Mesenchymal Stem/Stromal Cells, an Immune Privileged Niche for Viruses?. Int. J. Mol. Sci..

[13] Li F., Tang Y., Song B., Yu M., Li Q., Zhang C., Hou J., Yang R. (2019). Nomenclature Clarification: Synovial Fibroblasts and Synovial Mesenchymal Stem Cells. Stem Cell Res. Ther..

[14] Davidson S., Coles M., Thomas T., Kollias G., Ludewig B., Turley S., Brenner M., Buckley C.D. (2021). Fibroblasts as Immune Regulators in Infection, Inflammation and Cancer. Nat. Rev. Immunol..

[15] Payet M., Septembre-Malaterre A., Gasque P., Guillot X. (2023). Human Synovial Mesenchymal Stem Cells Expressed Immunoregulatory Factors IDO and TSG6 in a Context of Arthritis Mediated by Alphaviruses. Int. J. Mol. Sci..

[16] Payet M., Dargai F., Gasque P., Guillot X. (2021). Epigenetic Regulation (Including Micro-RNAs, DNA Methylation and Histone Modifications) of Rheumatoid Arthritis: A Systematic Review. Int. J. Mol. Sci..

[17] Bartel D.P. (2004). MicroRNAs: Genomics, Biogenesis, Mechanism, and Function. Cell.

[18] Najm A., Blanchard F., Le Goff B. (2019). Micro-RNAs in Inflammatory Arthritis: From Physiopathology to Diagnosis, Prognosis and Therapeutic Opportunities. Biochem. Pharmacol..

[19] Stanczyk J., Pedrioli D.M.L., Brentano F., Sanchez-Pernaute O., Kolling C., Gay R.E., Detmar M., Gay S., Kyburz D. (2008). Altered Expression of MicroRNA in Synovial Fibroblasts and Synovial Tissue in Rheumatoid Arthritis. Arthritis Rheum..

[20] Xie M., Wang J., Gong W., Xu H., Pan X., Chen Y., Ru S., Wang H., Chen X., Zhao Y. (2019). NF-κB-Driven miR-34a Impairs Treg/Th17 Balance via Targeting Foxp3. J. Autoimmun..

[21] Dang Q., Yang F., Lei H., Liu X., Yan M., Huang H., Fan X., Li Y. (2017). Inhibition of microRNA-34a Ameliorates Murine Collagen-Induced Arthritis. Exp. Ther. Med..

[22] Evangelatos G., Fragoulis G.E., Koulouri V., Lambrou G.I. (2019). MicroRNAs in Rheumatoid Arthritis: From Pathogenesis to Clinical Impact. Autoimmun. Rev..

[23] Liu W., Wu Y.-H., Zhang L., Xue B., Wang Y., Liu B., Liu X.-Y., Zuo F., Yang X.-Y., Chen F.-Y. (2018). MicroRNA-146a Suppresses Rheumatoid Arthritis Fibroblast-like Synoviocytes Proliferation and Inflammatory Responses by Inhibiting the TLR4/NF-kB Signaling. Oncotarget.

[24] Yang S., Yang Y. (2015). Downregulation of microRNA-221 Decreases Migration and Invasion in Fibroblast-like Synoviocytes in Rheumatoid Arthritis. Mol. Med. Rep..

[25] Wang Y., Feng T., Duan S., Shi Y., Li S., Zhang X., Zhang L. (2020). miR-155 Promotes Fibroblast-like Synoviocyte Proliferation and Inflammatory Cytokine Secretion in Rheumatoid Arthritis by Targeting FOXO3a. Exp. Ther. Med..

[26] Niederer F., Trenkmann M., Ospelt C., Karouzakis E., Neidhart M., Stanczyk J., Kolling C., Gay R.E., Detmar M., Gay S. (2012). Down-Regulation of microRNA-34a* in Rheumatoid Arthritis Synovial Fibroblasts Promotes Apoptosis Resistance. Arthritis Rheum..

[27] Chen Z., Wang H., Xia Y., Yan F., Lu Y. (2018). Therapeutic Potential of Mesenchymal Cell-Derived miRNA-150-5p-Expressing Exosomes in Rheumatoid Arthritis Mediated by the Modulation of MMP14 and VEGF. J. Immunol..

[28] Dobi A., Gasque P., Guiraud P., Selambarom J. (2021). Irinotecan (CPT-11) Canonical Anti-Cancer Drug Can Also Modulate Antiviral and Pro-Inflammatory Responses of Primary Human Synovial Fibroblasts. Cells.

[29] Hanna J., Ah-Pine F., Boina C., Bedoui Y., Gasque P., Septembre-Malaterre A. (2023). Deciphering the Role of the Anaphylatoxin C3a: A Key Function in Modulating the Tumor Microenvironment. Cancers.

[30] Septembre-Malaterre A., Boina C., Douanier A., Gasque P. (2022). Deciphering the Antifibrotic Property of Metformin. Cells.

[31] Lambert C., Morales-Sánchez P., García A.V., Villa-Fernández E., Latorre J., García-Villarino M., Turienzo Santos E.O., Suárez-Gutierrez L., Uría R.R., Navarro S.S. (2025). Exploring Differential miRNA Expression Profiles in Muscular and Visceral Adipose Tissue of Patients with Severe Obesity. Int. J. Obes..

[32] Baio R., Napodano G., Caruana C., Molisso G., Di Mauro U., Intilla O., Pane U., D’Angelo C., Francavilla A.B., Guarnaccia C. (2022). Association between Obesity and Frequency of High-Grade Prostate Cancer on Biopsy in Men: A Single-Center Retrospective Study. Mol. Clin. Oncol..

[33] Kastrinaki M.-C., Papadaki H.A. (2009). Mesenchymal Stromal Cells in Rheumatoid Arthritis: Biological Properties and Clinical Applications. Curr. Stem Cell Res. Ther..

[34] Waterman R.S., Tomchuck S.L., Henkle S.L., Betancourt A.M. (2010). A New Mesenchymal Stem Cell (MSC) Paradigm: Polarization into a Pro-Inflammatory MSC1 or an Immunosuppressive MSC2 Phenotype. PLoS ONE.

[35] Dorronsoro A., Fernández-Rueda J., Fechter K., Ferrin I., Salcedo J.M., Jakobsson E., Trigueros C. (2013). Human Mesenchymal Stromal Cell-Mediated Immunoregulation: Mechanisms of Action and Clinical Applications. Bone Marrow Res..

[36] Sokolova M.V., Schett G., Steffen U. (2022). Autoantibodies in Rheumatoid Arthritis: Historical Background and Novel Findings. Clin. Rev. Allergy Immunol..

[37] Li J., Wan Y., Guo Q., Zou L., Zhang J., Fang Y., Zhang J., Zhang J., Fu X., Liu H. (2010). Altered microRNA Expression Profile with miR-146a Upregulation in CD4+ T Cells from Patients with Rheumatoid Arthritis. Arthritis Res. Ther..

[38] Pandis I., Ospelt C., Karagianni N., Denis M.C., Reczko M., Camps C., Hatzigeorgiou A.G., Ragoussis J., Gay S., Kollias G. (2012). Identification of microRNA-221/222 and microRNA-323-3p Association with Rheumatoid Arthritis via Predictions Using the Human Tumour Necrosis Factor Transgenic Mouse Model. Ann. Rheum. Dis..

[39] Guo Q., Wang Y., Xu D., Nossent J., Pavlos N.J., Xu J. (2018). Rheumatoid Arthritis: Pathological Mechanisms and Modern Pharmacologic Therapies. Bone Res..

[40] Murata K., Yoshitomi H., Tanida S., Ishikawa M., Nishitani K., Ito H., Nakamura T. (2010). Plasma and Synovial Fluid microRNAs as Potential Biomarkers of Rheumatoid Arthritis and Osteoarthritis. Arthritis Res. Ther..

[41] Renman E., Brink M., Ärlestig L., Rantapää-Dahlqvist S., Lejon K. (2021). Dysregulated microRNA Expression in Rheumatoid Arthritis Families—A Comparison between Rheumatoid Arthritis Patients, Their First-Degree Relatives, and Healthy Controls. Clin. Rheumatol..

[42] Niimoto T., Nakasa T., Ishikawa M., Okuhara A., Izumi B., Deie M., Suzuki O., Adachi N., Ochi M. (2010). MicroRNA-146a Expresses in Interleukin-17 Producing T Cells in Rheumatoid Arthritis Patients. BMC Musculoskelet. Disord..

[43] Rezaeepoor M., Pourjafar M., Tahamoli-Roudsari A., Basiri Z., Hajilooi M., Solgi G. (2020). Altered Expression of microRNAs May Predict Therapeutic Response in Rheumatoid Arthritis Patients. Int. Immunopharmacol..

[44] Stanczyk J., Ospelt C., Karouzakis E., Filer A., Raza K., Kolling C., Gay R., Buckley C.D., Tak P.P., Gay S. (2011). Altered Expression of MicroRNA-203 in Rheumatoid Arthritis Synovial Fibroblasts and Its Role in Fibroblast Activation. Arthritis Rheum..

[45] Quero L., Tiaden A.N., Hanser E., Roux J., Laski A., Hall J., Kyburz D. (2019). miR-221-3p Drives the Shift of M2-Macrophages to a Pro-Inflammatory Function by Suppressing JAK3/STAT3 Activation. Front. Immunol..

[46] Koch A.E., Kunkel S.L., Harlow L.A., Johnson B., Evanoff H.L., Haines G.K., Burdick M.D., Pope R.M., Strieter R.M. (1992). Enhanced Production of Monocyte Chemoattractant Protein-1 in Rheumatoid Arthritis. J. Clin. Investig..

[47] Moon S.-J., Park M.-K., Oh H.-J., Lee S.-Y., Kwok S.-K., Cho M.-L., Ju J.H., Park K.-S., Kim H.-Y., Park S.-H. (2010). Engagement of Toll-like Receptor 3 Induces Vascular Endothelial Growth Factor and Interleukin-8 in Human Rheumatoid Synovial Fibroblasts. Korean J. Intern. Med..

[48] Maeda Y., Farina N.H., Matzelle M.M., Fanning P.J., Lian J.B., Gravallese E.M. (2017). Synovium-Derived MicroRNAs Regulate Bone Pathways in Rheumatoid Arthritis. J. Bone Miner. Res..

[49] Zhou Q., Haupt S., Kreuzer J.T., Hammitzsch A., Proft F., Neumann C., Leipe J., Witt M., Schulze-Koops H., Skapenko A. (2015). Decreased Expression of miR-146a and miR-155 Contributes to an Abnormal Treg Phenotype in Patients with Rheumatoid Arthritis. Ann. Rheum. Dis..

