# Supplementary material for: Overexpression of miR-146a-5p and miR-221-3p in Human Synovial MSC-like Cells Favoured the Expression of Pro-Inflammatory Mediators in an In Vitro Model of Rheumatoid Arthritis
Source: Cells. 2026 Apr 14;15(8):691. doi: 10.3390/cells15080691 (PMC13114388; doi:10.3390/cells15080691)
Supplement: Supplementary file 1 [file cells-15-00691-s001.zip › cells-4182760-supplementary.pdf]

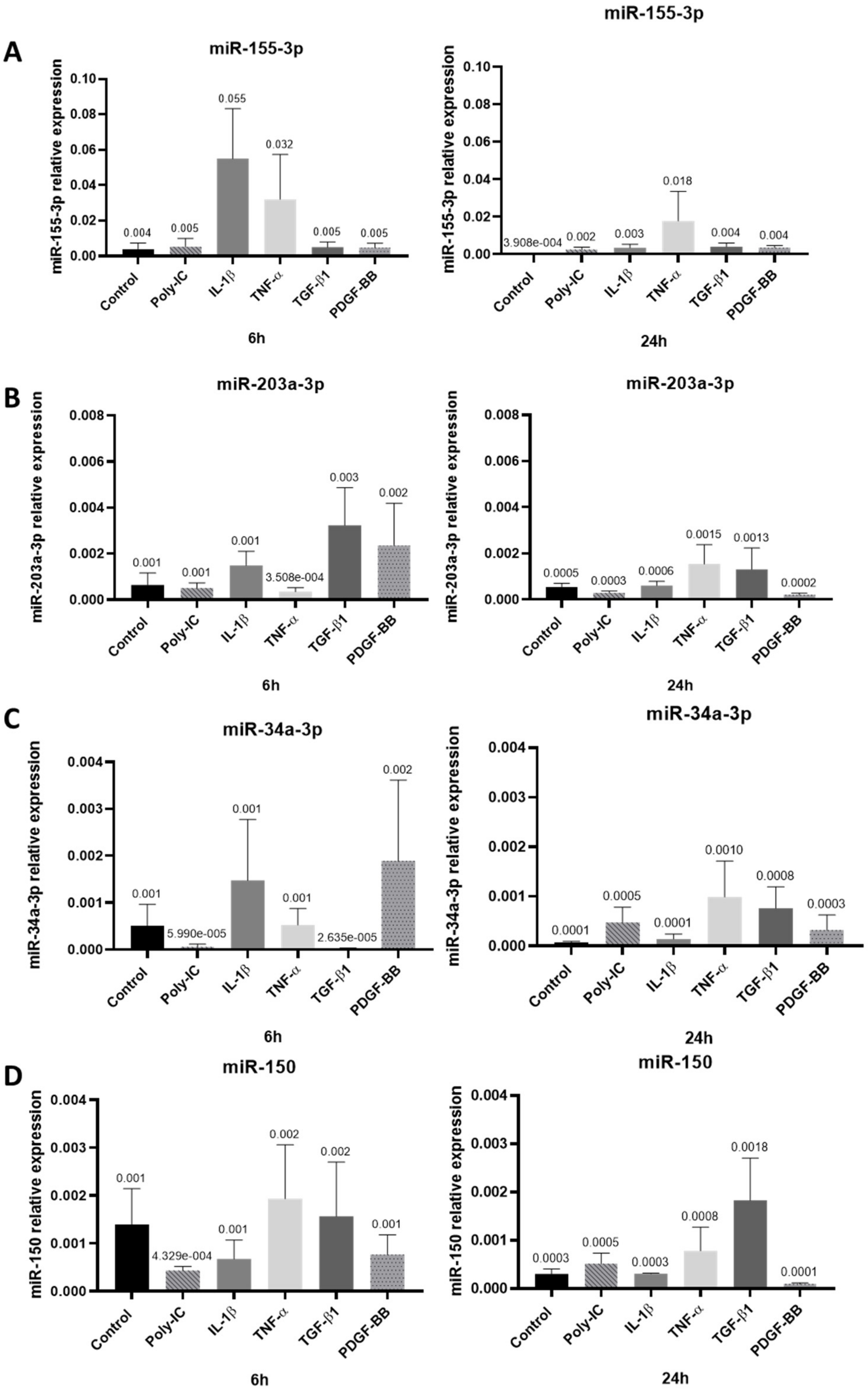

Figure S1: Pro-inflammatory mediators and growth factors had no effect on miR-155-3p, miR-203a-3p, miR-34a-3p and miR-150 expression. Human synovial tissue-derived MSC were stimulated with poly I:C (10 µg/ml), IL-1β, TNF-α, IFN-γ, TGFβ1 and PDGFBB (20 ng/ml) during 6h and 24h. qPCR was performed to determine the expression of (A) miR-155-3p, (B) miR-203a-3p, (C) miR-34a-3p and (D) miR-150. Reported values are mean ± SEM.

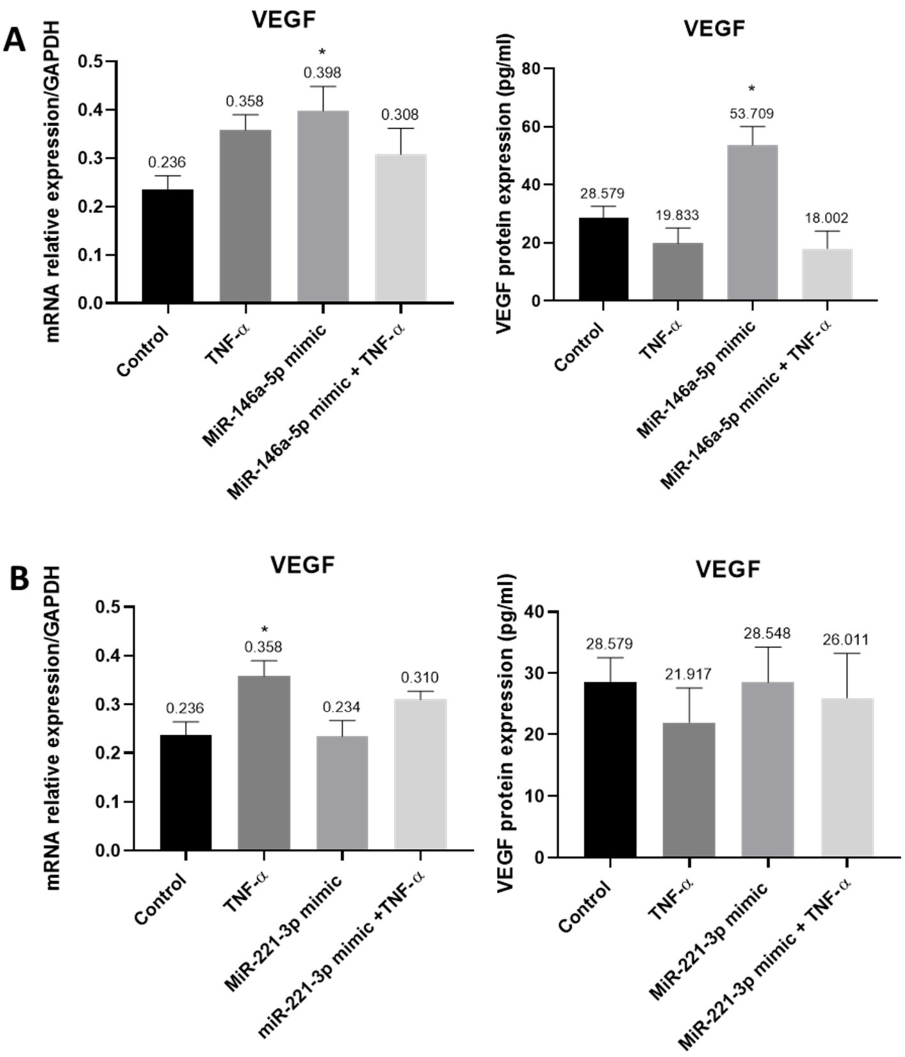

Figure S2: miR-146a-5p upregulated VEGF expression. Synovial tissue derived-MSC were transfected with miR-146a-5p mimic or miR-221-3p mimic (100 nM) using lipofectamine 3000 or non-transfected (+ lipofectamine) during 48h. Then, transfected cells were stimulated with TNF-α during 6h, non-transfected cells were not stimulated (control) or stimulated with TNF-α during 6h. mRNA and protein expression of VEGF in synovial tissue-derived MSC (A) transfected with miR-146a-5p or (B) miR-221-3p was determined by qRTPCR and ELISA test. Reported values are mean ± SEM and p-value was calculated using Kruskal-Wallis test followed by Dunn's post hoc test: *: $p<0.05$ as compared to control (non-transfected cells). Experiments were performed in five independent experiments.

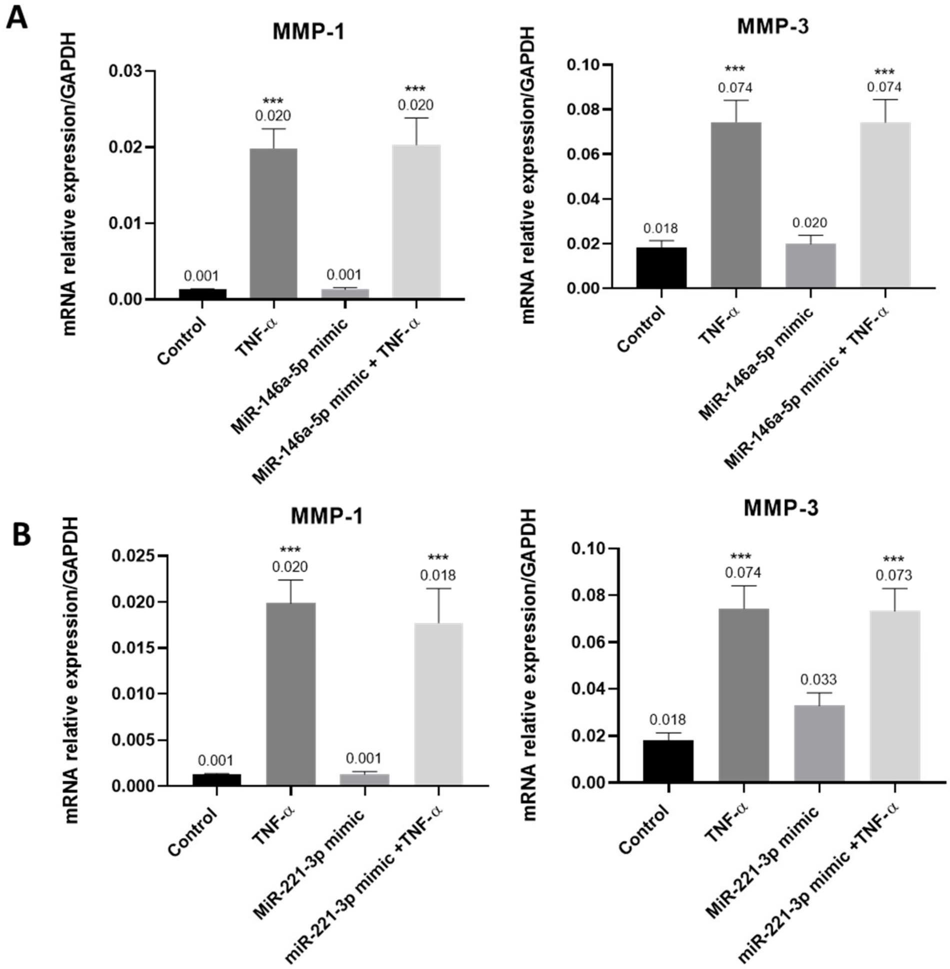

Figure S3: MiR-146a-5p and miR-221-3p didn't affect the expression of MMPs in synovial tissue-derived MSC stimulated with TNF-α. Synovial tissue derived-MSC were transfected with miR-146a-5p mimic or miR-221-3p mimic (100 nM) using lipofectamine 3000 or non-transfected (+ lipofectamine) during 48h. Then, transfected cells were stimulated with TNF-α during 6h, non-transfected cells were not stimulated (control) or stimulated with TNF-α during 6h. mRNA and protein expression of MMP-1 and MMP-3 in synovial tissue-derived MSC (A) transfected with miR-146a-5p or (B) miR-221-3p was determined by qRTPCR and ELISA test. Reported values are mean ± SEM and p-value was calculated using ANOVA Bonferroni test: ***: $p<0.001$ as compared to control (non-transfected cells). Experiments were performed in five independent experiments.

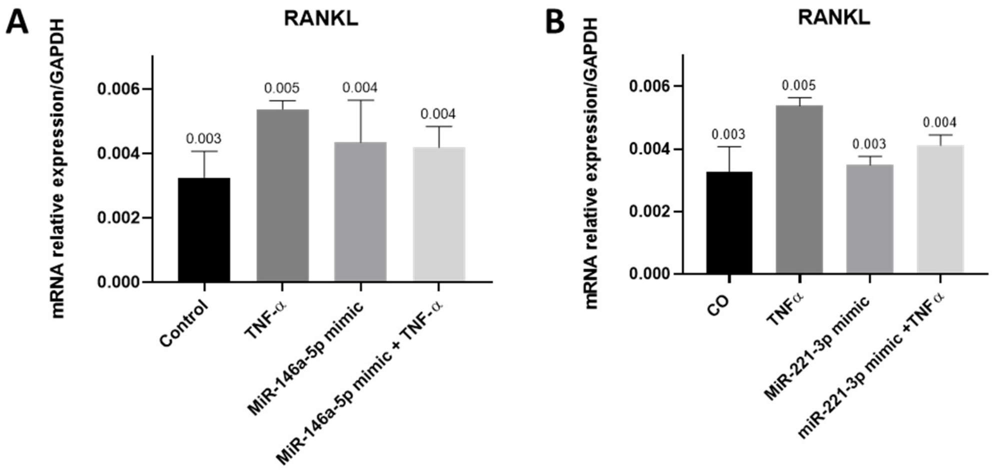

Figure S4: MiR-146a-5p and miR-221-3p did not affect RANKL mRNA expression in synovial tissue-derived MSC. Synovial tissue derived-MSC were transfected with miR-146a-5p mimic or miR-221-3p mimic (100 nM) using lipofectamine 3000 or non-transfected (+ lipofectamine) during 48h. Then, transfected cells were stimulated with TNF-α during 6h, non-transfected cells were not stimulated (control) or stimulated with TNF-α during 6h. mRNA expression of RANKL in synovial tissue-derived MSC (A) transfected with miR-146a-5p or (B) miR-221-3p was determined by qRTPCR and ELISA test. Reported values are mean ± SEM and p-value was calculated using ANOVA Bonferroni test. Experiments were performed in five independent experiments.

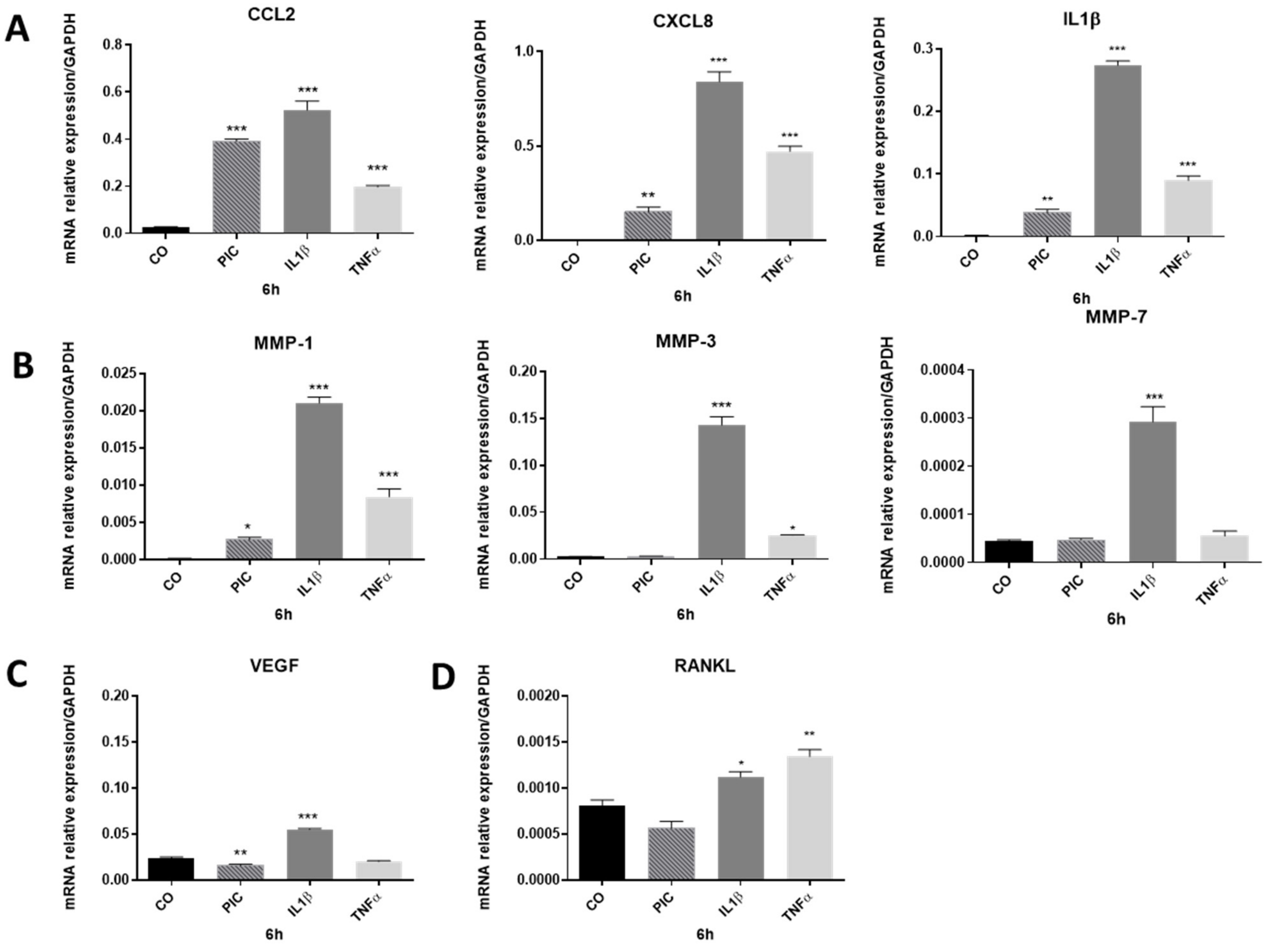

Figure S5: Expression of pro-inflammatory mediators, MMPs, VEGF and RANL in response to pro-inflammatory stimuli. Synovial tissue derived-MSC were stimulated with poly I:C (10 µg/ml), IL-1β and TNF-α (20 ng/ml). mRNA expression of pro-inflammatory mediators (A), MMPs (B), VEGF (C) and RANKL (D) was determined by qRTPCR. Reported values are mean ± SEM and p-value was calculated using ANOVA Bonferroni test: *: p<0,05, **: p<0,01, ***: p<0.001 as compared to control (unstimulated cells).
